# Photodynamic therapy as a novel treatment for halitosis in adolescents: study protocol for a randomized controlled trial

**DOI:** 10.1186/1745-6215-15-443

**Published:** 2014-11-14

**Authors:** Rubia Garcia Lopes, Camila Haddad Leal de Godoy, Alessandro Melo Deana, Maria Eugenia Simões Onofre de Santi, Renato Araujo Prates, Cristiane Miranda França, Kristianne Porta Santos Fernandes, Raquel Agnelli Mesquita-Ferrari, Sandra Kalil Bussadori

**Affiliations:** University Nove de Julho, Rua Vergueiro, 235, Liberdade, São Paulo, SP 01504-000 Brazil

**Keywords:** Halitosis, Photodynamic therapy, Adolescent

## Abstract

**Background:**

Halitosis is a common problem that affects a large portion of the population worldwide. The origin of this condition is oral in 90% and systemic in 10% of cases. The unpleasant odor is mainly the result of volatile sulfur compounds produced by Gram-negative bacteria. However, it has recently been found that anaerobic Gram-positive bacteria also produce hydrogen sulfide (H_2_S) in the presence of amino acids, such as cysteine. Light, both with and without the use of chemical agents, has been used to induce therapeutic and antimicrobial effects. In photodynamic therapy, the antimicrobial effect is confined to areas covered by photosensitizing dye. The aim of the present study is to evaluate the antimicrobial effect of photodynamic therapy on halitosis in adolescents through the analysis of volatile sulfur compounds measured using gas chromatography and microbiological analysis of coated tongue.

**Methods/Design:**

A quantitative clinical trial will be carried out involving 60 adolescents randomly divided into the following groups: group 1 will receive treatment with a tongue scraper, group 2 will receive photodynamic therapy applied to the posterior two-thirds of the dorsum of the tongue, and group 3 will receive combined treatment (tongue scraper and photodynamic therapy). Gas chromatography (OralChroma^TM^) and microbiological analysis will be used for the diagnosis of halitosis at the beginning of the study. Post-treatment evaluations will be conducted at one hour and 24 hours after treatment. The statistical analysis will include the Shapiro-Wilk test for the determination of the distribution of the data. If normal distribution is demonstrated, analysis of variance followed by Tukey’s test will be used to compare groups. The Kruskal-Wallis test followed by the Student-Newman-Keuls test will be used for data with non-normal distribution. Either the paired *t*-test or the Wilcoxon test will be used to compare data before and after treatment, depending on the distribution of the data.

**Discussion:**

The results of this trial will determine the efficacy of using photodynamic therapy alone or in combination with a tongue scraper to treat bad breath in adolescents.

**Trial registration:**

The protocol for this study was registered with Clinical Trials (registration number NCT02007993) on 10 December 2013.

## Background

Halitosis is a term used to define an unpleasant odor that emanates from the mouth, stemming from either a local or systemic origin [1–3]. This common problem affects a large portion of the population worldwide and causes considerable embarrassment. Therefore, halitosis has a negative impact on social communication and quality of life [4]. The lack of standardization in the protocol for the diagnosis and treatment of halitosis hinders the comparison of data from epidemiological studies conducted in different countries and yet it is believed that 25% of the population are affected by this condition [5].

Studies on the etiology of halitosis report that 2% of cases stem from renal, metabolic, hepatic, endocrinological and gastrointestinal disorders (such as infection by *Helicobacter pylori* and intestinal blockage), and 8% are due to conditions of the respiratory system and conditions of the ears, nose and throat (ENT), such as acute tonsillitis, postnasal drip, sinusitis and tonsillolith. The majority of cases (80 to 90%) are directly linked to conditions of the oral cavity, such as periodontal disease, coated tongue, poor oral hygiene, salivary abnormalities (change in pH and hyposialy), stomatitis, intra-oral neoplasm, pulp exposure, extraction wounds and crowding of the teeth [5–8].

Bad breath mainly stems from volatile sulfur compounds (VSCs) produced by the action of anaerobic Gram-negative bacteria (*Fusobacterium nucleatum*, *Selenomonas*, *Treponema denticola*, *Prevotella intermedia*, *Tannerella forsythyia*, *Porphyromonas gingivalis*, *Bacteroides forsythus* and *Eubacterium*) found in the oral cavity on substrates containing sulfur [9–11]. The VSCs produced by the metabolism of these bacteria are hydrogen sulfide (H_2_S), found mainly on the dorsum of the tongue, methanethiol (CH_3_SH) in gingival pockets and dimethyl sulfide (CH_3_SCH_3_), which has an extra-oral origin [12–15]. The concentration of these compounds is used as an indicator of halitosis [3, 16].

Recently, the anaerobic Gram-positive bacterium *Solobacterium moorei* (also known as *Bulleidia moorei*) has been associated with halitosis due to the production of H_2_S in the presence of different supplements containing amino acids, especially cysteine [17, 18]. Studies have demonstrated that the presence of these bacteria on the dorsum of the tongue, as well as in saliva and periodontal pockets, can lead to both halitosis and systemic problems such as complications during pregnancy, cardiovascular disease and chronic lower respiratory infection [19], which is considered the third most common cause of death [2, 20–23].

### Detection

Two main methods are used to evaluate halitosis: a subjective (organoleptic) evaluation and an objective evaluation (quantitative measure of VSCs, gas chromatography (GC) and monitor analysis) [24–27]. Studies comparing the efficacy of these methods report GC to be the most objective and efficacious method for the individual detection of H_2_S, CH_3_SH and CH_3_SCH_3_, allowing the evaluation of both the intensity of bad breath and its origin [5, 15]. Indeed, GC is currently considered the gold standard for the detection of halitosis [11]. However, the majority of researchers have used a combination of both subjective and objective evaluations, whereas others have only used an organoleptic evaluation due to its ease of execution and low cost [24]. Halitosis can be analyzed using a sulfide monitor, such as the Halimeter (Interscan Corporation, Chatsworth, California, United States) [3, 4, 26–28], which determines the total amount of VSCs in parts per billion (ppb) under normal conditions.

### Photodynamic therapy

Photodynamic therapy (PDT) was discovered in 1900 by Oskar Raab and Hermann von Tappeiner. In the 1970s, PDT began to be used for the treatment of cancer. Recently, antimicrobial PDT has been used as a treatment option for localized infections [29]. PDT involves the use of a non-toxic light-sensitive photosensitizer combined with visible light at the appropriate wavelength to coincide with the absorption spectrum of the photosensitizer, which reaches a state of excitation after absorbing the photons, reacting with the oxygen in the medium to form reactive oxygen species. This phototoxic reaction induces the destruction of bacterial cells. The antimicrobial effect is confined to areas covered by the light-activated photosensitizer, quickly acting on the target organisms when the appropriate energy dose and output power are used [9, 29–33]. According to Wainwright [34], bacterial resistance to PDT is unlikely, as the singlet oxygen and free radicals formed interact with different bacterial cell structures and different metabolic pathways [32, 33].

The conventional treatment of halitosis related to oral conditions consists of the chemical reduction of microorganisms with a mouthwash, such as 0.2% chlorhexidine, essential oils, triclosan and hydrogen peroxide, the mechanical removal of nutrients with a tongue scraper or brush, the masking of odor with chewing gum, mints and breath spray, and the transformation of VSCs using zinc plus chlorhexidine [2, 5, 10, 12, 35–37]. However, the irregular characteristics of the surface of the dorsum of the tongue make the adequate reduction in bacterial a particular challenge [2, 36, 38].

Considering the scarcity of studies addressing the effect of PDT on tongue biofilm, the aim of the present study was to evaluate the effectiveness of PDT on the dorsum of the tongue in adolescents with halitosis by an analysis of VSCs and microbiological analysis of the tongue.

## Methods/Design

This study will be carried out in compliance with regulatory norms governing research involving human subjects. Approval was obtained from the Human Research Ethics Committee of University Nove de Julho (Brazil) under process number 037315/2013, and the study is registered with the United States National Institutes of Health (Clinical Trials.gov registration number: NCT02007993). The guardians of the participants will be informed regarding the procedures and will sign a statement of informed consent authorizing the participation of their sons and daughters in compliance with Resolution 196/96 of the Brazilian National Health Board.

**Figure 1:**
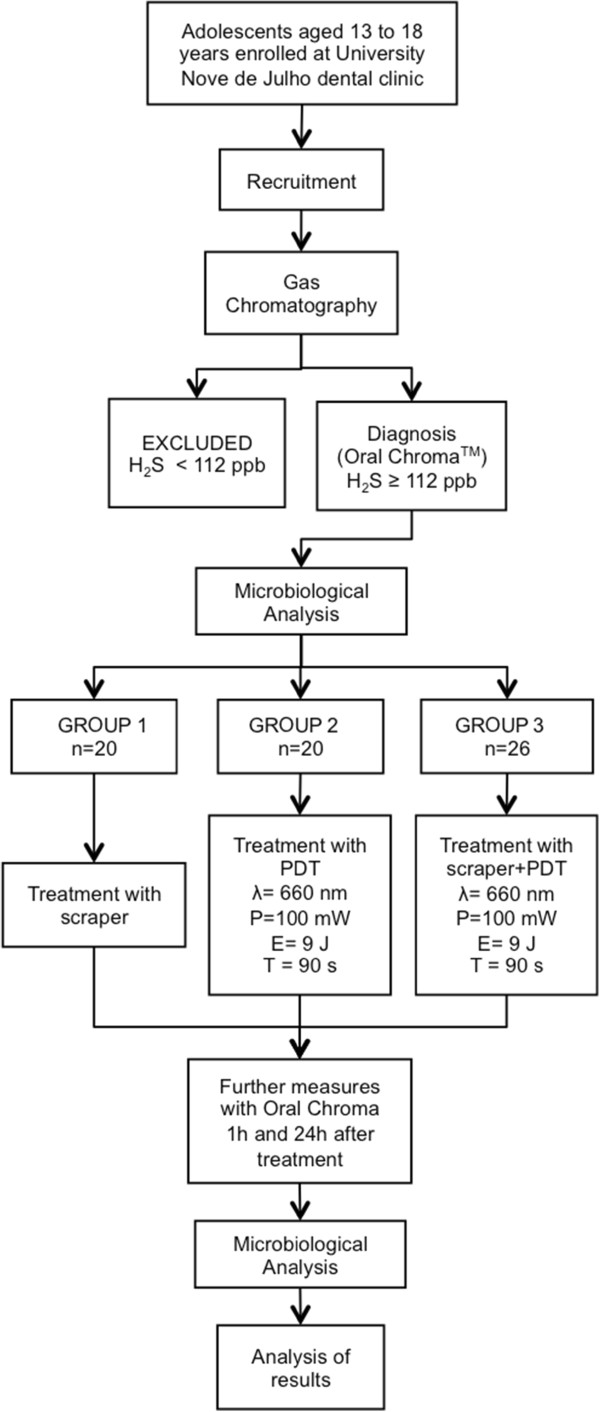
**Flowchart of study.** P = output power; E = energy and T = time in seconds.

Male and female adolescents enrolled at the dental clinic of the university will be recruited for the study. Those aged between 13 and 18 years, with a diagnosis of halitosis and OralChroma™ results of H_2_S ≥112 ppb during the cysteine challenge [11, 15, 39, 40] will be included. The exclusion criteria will be [41] dentofacial anomalies, currently undergoing orthodontic or orthopedic treatment, current use of a removable appliance, implant or dentures, periodontal disease, teeth with carious lesions, currently undergoing cancer treatment, diabetes mellitus, systemic (gastrointestinal, renal or hepatic disorder) conditions, ear, nose or throat conditions, respiratory conditions, antibiotic therapy in the previous month, current pregnancy [5] or hypersensitivity to the photosensitizer. As this is a randomized clinical trial, the recommendations of the Consolidated Standards of Reporting Trials (CONSORT) will be used to ensure greater transparency and quality (Figure 1).

The subjects selected will be randomly allocated to three groups (Table 1). All individuals will be submitted to evaluations with OralChroma™ before and after treatment.

**Table 1:** **Summary of experimental conditions**

Group	Halitosis	Treatment	
1	H_2_S ≥112 ppb	Tongue scraper	
2	H_2_S ≥112 ppb	PDT	
		E = 9 J	T = 90 s
3	SH_2_ ≥ 112 ppb	Tongue scraper + PDT	
		E = 9 J	T = 90 s

H_2_S = hydrogen sulfide; ppb = part per billion; PDT = photodynamic therapy; E = energy; T = time in seconds.

### Microbiological analysis

Microbiological analyses of coated tongue will be performed before and after treatment using a 1-μl inoculation loop for the collection of biofilm samples from the dorsum of the tongue. The samples will be transferred to 1.5-ml vials with reduced transport fluid and placed in a vortex mixer (Prolab, São Paulo – Brazil) for approximately 30 seconds for homogenization. Ten-fold serial dilution will be prepared in 180 μl of sterile phosphate buffered saline (Probac, São Paulo – Brazil) and aliquots of 10^−2^, 10^−3^, 10^−4^ and 10^−5^ will be transferred to plates with brain-heart infusion agar (Probac, São Paulo – Brazil). As the main bacteria responsible for the production of VSCs are Gram-negative, the plates will be incubated in anaerobic jar for 72 hours at 37°C, following by the quantification of colony-forming units [10, 42].

### Halitosis detection

The literature describes a number of methods for measuring halitosis, such as an organoleptic evaluation of the air emanating from the oral cavity [16, 26] using a sulfide monitor [16, 25, 43] or GC [11, 43, 44]. However, it has been demonstrated that the organoleptic test can be influenced by the olfactory capacity and emotional state of the examiner, as well as climatic conditions [3]. Therefore, the portable OralChroma™ device Abilit Corporation, Chuo-ku, Osaka - Japan) will be employed. This device uses a highly sensitive gas semiconductor sensor.

**Figure 2:**
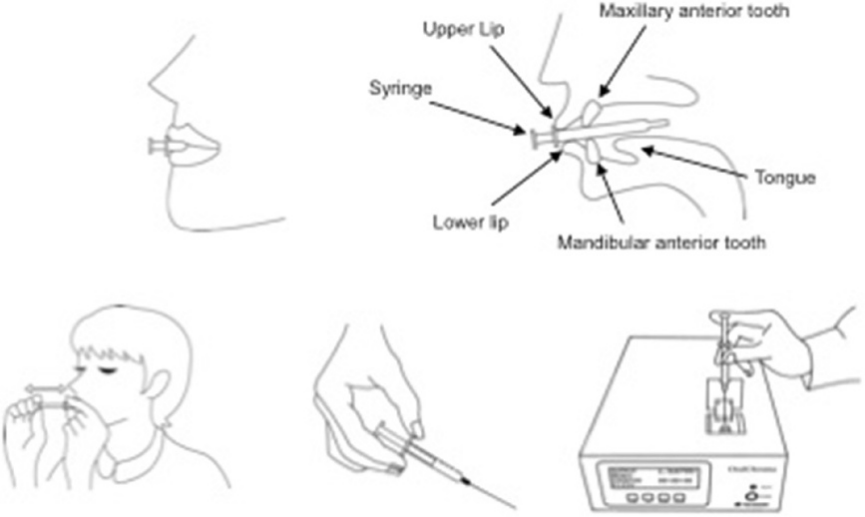
**Process for the acquisition of the sample for the halimetric.** OralChroma (Abiliti Corporation, Chuo-ku, Osaka – Japan). The OralChroma™ will be connected to the computer with a specific software program that allows the creation of a graph corresponding to the peaks and concentrations of VSCs (0 to 2913 ppb) with considerable precision after eight minutes. The results are stored in the program, as well as in the device itself, and can be retrieved at any time for comparisons of the readings before, during and after treatment.

The participant will first rinse with cysteine (Fórmula & Ação, São Paulo – Brazil) for one minute (cysteine 10 mM - 16 mg of cysteine in 100 ml of distilled water - 16% mg). A syringe will be placed in the participant’s mouth with the plunger completely inserted. The participant will close his or her mouth, breathe through the nose and remain still with the mouth closed for one minute. The participant will be instructed not to touch the tip of the syringe with his or her tongue. The plunger will then be withdrawn, pushed back in to empty the air into the participant’s mouth and will be withdrawn again to fill the syringe with the breath sample. The tip of the syringe will be cleaned to remove saliva and a gas injection needle will be placed on the syringe. The plunger will be adjusted to 0.5 ml and the contents will be injected into the input of the device in a single motion (Figure 2) [15].

### Analysis of VSCs

OralChroma™ (Abilit Corporation) was developed in Japan for the individual determination of H_2_S, CH_3_SH and CH_3_SCH_3_, allowing for the evaluation of both the intensity of bad breath and its origin [5, 11, 15]. H_2_S originates mainly from bacteria on the dorsum of the tongue. Values greater than 112 ppb indicate halitosis. CH_3_SH is found in greater concentration in periodontal pockets. Values up to 26 ppb are considered normal. Periodontal disease typically results in a high CH_3_SH:H_2_S ratio (>3:1). CH_3_SCH_3_ may have a periodontal or systemic (intestine, liver or lung) origin and may also be temporarily caused by the ingestion of certain foods and beverages. The distinction between CH_3_SCH_3_ of an oral or systemic origin can be made through the comparison of the results of the halimetric (OralChroma™) with and without a cysteine challenge. The perception threshold for CH_3_SCH_3_ is very low (8 ppb). Other non-VSC odors may appear in a peak prior to the theoretical first peak, which is H_2_S [15].

To maximize the standardization of the readings, the exam will be carried out in the morning and the participants will be instructed to avoid the ingestion of foods with garlic, onion or strong spices, as well as the consumption of alcohol and the use of an antiseptic mouthwash. On the morning of the exam, more than two hours should have passed since any food intake and the participants are to abstain from coffee, hard candy, chewing gum, oral hygiene products and personal care items containing fragrances (aftershave, deodorant, perfume and creams). Brushing will be performed with water alone [27, 45].

### Photodynamic therapy

The THERAPY XT-ES™ (DMC ABC Medical and Dental Equipment, São Paulo, Brazil) with a red (660 nm) and infrared (810 nm) laser and a fine tip (for regions of difficult access) will be used. Only the volunteer and operator will be present at the time of PDT and both will be wearing protective eyewear. The active point of the laser will be covered with disposable clear plastic wrap (PVC) for hygiene purposes and to avoid cross-contamination. The operator will use the appropriate clothing.

**Figure 3:**
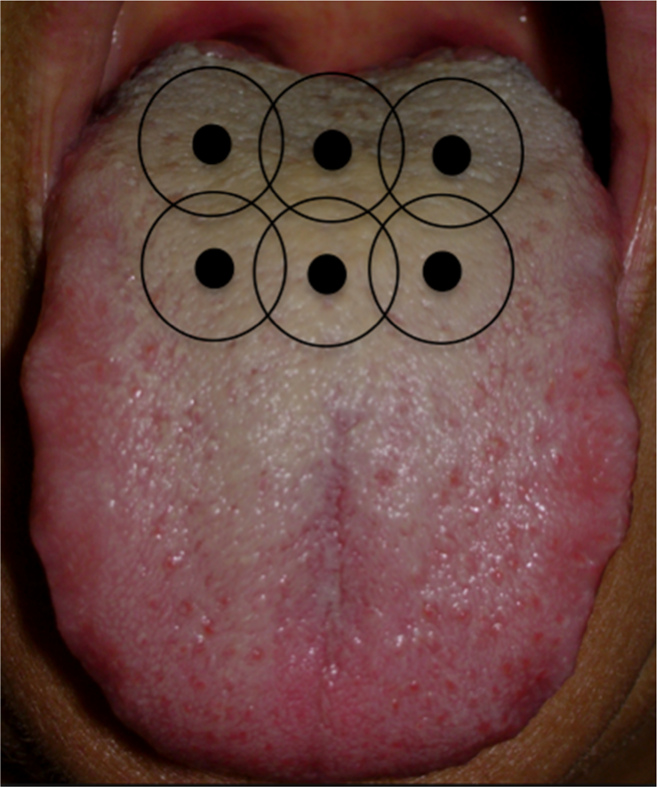
**Points of photodynamic therapy application.**

A single session of PDT will be performed with the Chimiolux™ methylene blue photosensitizer (DMC ABC Medical and Dental Equipment, São Paulo, Brazil) at a concentration of 0.005% (165 μm) applied to the middle and posterior thirds of the dorsum of the tongue. After five minutes of pre-irradiation time for incubation, the excess will be removed with an aspirator to maintain the surface moist with the photosensitizer alone (without the use of water). A total of six points will be irradiated (Figure 3). Based on studies developed for the treatment of periodontal disease with PDT [46–52] and a previous pilot study [53], the device will be calibrated with a wavelength of 660 nm, power output of 100 mW, fluency of 320 J/cm^2^, irradiance of 3537 mW/cm^2^ and an energy dose of 9 joules for 90 seconds per point in groups 2 and 3. The punctual application method will be used with the conventional tip in contact with the tongue (Table 2).

**Table 2:** **Parameters of laser**

Parameter	Red laser
Center wavelength (nm)	660
Spectral bandwidth (FWHM) (nm)	5
Operating mode	Continuous wave
Average radiant power (mW)	100
Polarization	Random
Aperture diameter (cm)	0.094
Irradiance at aperture (mW/cm^2^)	3537
Beam profile	Multimode
Beam spot size at target (cm^2^)	0.02827
Irradiance at target (mW/cm^2^)	3537
Exposure duration (s)	90/120
Radiant exposure (J/cm^2^)	320/428
Radiant energy (J)	9/12
Number of points irradiated	9
Area irradiated (cm^2^)	0.254
Application technique	Contact
Number and frequency of treatment sessions	1 session
Total radiant energy (J)	81/108

### Tongue scraping intervention

**Figure 4:**
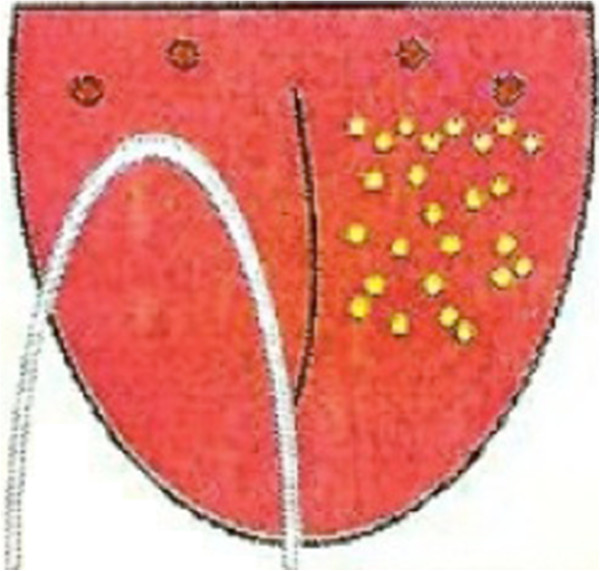
**Diagram of tongue scraper use.**

A Halicare™ tongue scraper (Odomed, São Paulo, Brazil) will be used for the removal of biofilm. The participant will be instructed to divide the tongue into two parts and scrape each side 10 times (Figure 4).

### Calculation of sample size

The error was established as , in which  and  are the means of groups 1 and 2. Assuming both samples as having the same size (n_1_ = n_2_), the sample size is obtained from the following equation:
1

in which  and  are the variances in groups 1 and 2, respectively. As more than two groups will be studied, the decision was made to employ the largest error found in the literature [54] to estimate the sample size. Assuming all groups as having normal or approximately normal distribution, and that the sample will be large enough for a significance level of α = 0.05, the *Z* value was determined to be 1.96. However, for the sample size, the test power was established as 1-β = 0.80. In case the hypothesis of normality in the samples was rejected, the sample size was corrected by 5%. Based on the experimental groups in the study by Tsai *et al*. [54] it was determined that each group should contain 26 participants (n = 26).

### Outcome measures

Only participants with H_2_S ≥112 ppb, CH_3_SH ≤26 ppb and CH_3_SCH_3_ ≤ 8 ppb will be part of the research, which limits participation to individuals with halitosis caused by coated tongue alone. Immediately after treatment, a second halimetry test will be performed and the results will be analyzed.

### Hypothesis

Our null hypothesis is that there will be no change in halitosis following the use of PDT. Our experimental hypothesis is that there will be a reduction in halitosis following the use of PDT alone or in combination with a tongue scraper.

### Organization and statistical analysis of data

The Shapiro-Wilk test will be used to determine the distribution of the halimetricdata. If the data presents with normal distribution, analysis of variance (ANOVA) followed by the Tukey test will be used to evaluate the correlation between each of the proposed treatments and halitosis. The paired *t*-test will be used to compare the data before and after each treatment and determine whether the treatments reduced the degree of halitosis. The Kruskal-Wallis test followed by the Student-Newman-Keuls test will be used for data with non-normal distribution, and the Wilcoxon test will be used to analyze the data before and after each treatment. Microbiological data presents with log-normal distribution and will therefore be analyzed using the methods described for data with normal distribution. A significance level of α = 0.05 will be used.

## Discussion

The main objective of the proposed study is to evaluate the effect of PDT with and without the use of a tongue scraper for the treatment of halitosis in adolescents. This objective has two aspects: the evaluation of VSC levels before and after treatment through a quantitative analysis of H_2_S using GC, and a microbiological analysis of the effect of PDT on coated tongue. The findings are expected to provide convincing evidence that PDT is more effective for the treatment of halitosis.

In the literature, the treatment of halitosis is performed using a tongue scraper with or without a mouthwash [2], which leads to a small, long-term reduction in the amount of bacteria on the tongue [38]. Thus, daily oral hygiene is needed to maintain a low level of bacterial proliferation. As the penetration of light and spreading of the photosensitizer do not seem to be affected by the posterior papillae of the tongue, treatment with PDT is promising and may achieve satisfactory results, especially when combined with conventional treatment.

## Trial status

The authors are currently recruiting participants. It begun on March of 2014 and we pretend to go until August of 2015.

## Abbreviations

CH_3_SCH_3_: Dimethyl sulfide
CH_3_SH: Methanethiol
GC: Gas chromatography
H_2_S: Hydrogen sulfide
PDT: Photodynamic therapy
VSC: Volatile sulfur compounds
